# Disease spectrum and long-term prognosis of patients with *BAG3*-associated neuromuscular diseases in Europe

**DOI:** 10.1093/brain/awaf223

**Published:** 2025-06-10

**Authors:** Gorka Fernández-Eulate, Cyril Gitiaux, Simone Thiele, Heinz Jungbluth, Anna Potulska-Chromik, Chiara Marini-Bettolo, Jean Baptiste Davion, Germán Morís, Eduard Gallardo, Montse Olivé, Carlos Pablo de Fuenmayor-Fernández de la Hoz, Frederique Audic, Arnaud Isapof, Maggie C Walter, Corrado Angelini, Enrico Bertini, Ulrike Schara-Schmidt, Kristl G Claeys, Maike F Dohrn, Mohamed Dembele, Frédéric Fer, Guy Brochier, Teresinha Evangelista, Anna Kostera-Pruszczyk, Shahram Attarian, Volker Straub, Cristina Domínguez-González, John Vissing, Pascale Richard, Corinne Metay, Diala Khraiche, Karim Wahbi, Tanya Stojkovic

**Affiliations:** Nord/Est/Ile-de-France Neuromuscular Diseases Reference Centre, Department of Neuro-Myology, Myology Institute, Pitié-Salpêtrière Hospital, Sorbonne Université, APHP, Paris 75013, France; Clinical Neurophysiology Unit, Neuromuscular Diseases Reference Centre, Necker Enfants Malades Hospital, APHP, Paris 75015, France; Friedrich-Baur-Institute, Department of Neurology, LMU University Hospital, LMU Munich, Munich D-80336, Germany; Department of Paediatric Neurology, Neuromuscular Service, Evelina’s Children Hospital, Guy’s & St. Thomas’ Hospital NHS Foundation Trust, London SE1 7EH, UK; Randall Centre for Cell and Molecular Biophysics, Muscle Signalling Section, Faculty of Life Sciences and Medicine (FoLSM), King’s College London, London SE1 9RT, UK; Department of Neurology, Medical University of Warsaw, Warsaw 02-019, Poland; The John Walton Muscular Dystrophy Research Centre, Newcastle University and Newcastle Hospitals NHS Foundation Trust, Newcastle-upon-Tyne NE1 3BZ, UK; Reference Centre for Neuromuscular Diseases Nord/Est/Ile-de-France, CHU Lille, Lille 59000, France; Department of Neurology, Neuromuscular Diseases Unit, Hospital Universitario Central de Asturias, Oviedo 33011, Spain; Department of Neurology, Neuromuscular Diseases Unit, Institut de Recerca Hospital de la Santa Creu i Sant Pau, Barcelona 08041, Spain; Biomedical Network Research Centre on Rare Diseases (CIBERER), Madrid 28029, Spain; Department of Neurology, Neuromuscular Diseases Unit, Institut de Recerca Hospital de la Santa Creu i Sant Pau, Barcelona 08041, Spain; Biomedical Network Research Centre on Rare Diseases (CIBERER), Madrid 28029, Spain; Department of Neurology, Neuromuscular Unit, Hospital Universitario 12 de Octubre, Research Institute imas12, Biomedical Network Research Centre on Rare Diseases (CIBERER), Instituto de Salud Carlos III, Madrid 28041, Spain; Neuropaediatric Department, Neuromuscular Diseases Reference Centre PACARARE, Timone Children’s Hospital, APHM, Marseille 13005, France; Neuropaediatric Department, Neuromuscular Diseases Reference Centre, Armand Trousseau Hospital, APHP, Paris 75012, France; Friedrich-Baur-Institute, Department of Neurology, LMU University Hospital, LMU Munich, Munich D-80336, Germany; Department of Neurosciences, University of Padua, Padua 35128, Italy; Research Unit of Neuromuscular and Neurodegenerative Disorders, Bambino Gesù Children’s Research Hospital, IRCCS, Rome 00146, Italy; Department of Neuropediatrics and Neuromuscular Centre for Children and Adolescents, Centre for Translational Neuro- and Behavioural Sciences, University of Duisburg-Essen, Essen D-45147, Germany; Department of Neurology, University Hospitals Leuven, Leuven 3000, Belgium; Department of Neurosciences, Laboratory for Muscle Diseases and Neuropathies, KU Leuven and Leuven Brain Institute (LBI), Leuven 3000, Belgium; Department of Neurology, Medical Faculty of the RWTH Aachen University, Uniklinik Aachen, Aachen 52074, Germany; AFM-Téléthon, Évry-Courcouronnes 91002, France; MYOdata Plateforme, Myology Institute, Paris 75013, France; Neuromuscular Morphology Unit, Myology Institute, Pitié-Salpêtrière Hospital, Paris 75013, France; Nord/Est/Ile-de-France Neuromuscular Diseases Reference Centre, Department of Neuro-Myology, Myology Institute, Pitié-Salpêtrière Hospital, Sorbonne Université, APHP, Paris 75013, France; Neuromuscular Morphology Unit, Myology Institute, Pitié-Salpêtrière Hospital, Paris 75013, France; Department of Neurology, Medical University of Warsaw, Warsaw 02-019, Poland; Neuromuscular Diseases and ALS Reference Centre, FILNEMUS, CHU La Timone, APHM, Marseille 13005, France; The John Walton Muscular Dystrophy Research Centre, Newcastle University and Newcastle Hospitals NHS Foundation Trust, Newcastle-upon-Tyne NE1 3BZ, UK; Department of Neurology, Neuromuscular Unit, Hospital Universitario 12 de Octubre, Research Institute imas12, Biomedical Network Research Centre on Rare Diseases (CIBERER), Instituto de Salud Carlos III, Madrid 28041, Spain; Copenhagen Neuromuscular Centre, Rigshospitalet Copenhagen University Hospital, Copenhagen DK-2100, Denmark; APHP, DMU BioGeMH-Sorbonne Université, Functional Unit of Molecular and Cellular Cardiogenetics and Myogenetics, Genetic Center of Pitié-Salpêtrière, INSERM UMRS 1166, Pitié-Salpêtrière Hospital, Paris 75013, France; Molecular and Cellular Cardiogenetics and Myogenetics Functional Unit, INSERM UMRS 974, Myology Institute, Pitié-Salpêtrière Hospital, APHP, Sorbonne Université, Paris 75013, France; Paediatric Cardiology Department, Necker-Enfants Malades Hospital, APHP, Paris 75015, France; Université Paris Cité, Imagine, Paris Cardiomyopathy Center Associated Laboratory, Paris 75015, France; APHP, Cochin Hospital, Cardiology Department, FILNEMUS, Centre de Référence des maladies Neuromusculaires Nord-Est-Ile de France, Paris Cité Université, Paris 75006, France; Nord/Est/Ile-de-France Neuromuscular Diseases Reference Centre, Department of Neuro-Myology, Myology Institute, Pitié-Salpêtrière Hospital, Sorbonne Université, APHP, Paris 75013, France

**Keywords:** BAG3, neuromyopathy, cardiomyopathy, autophagy

## Abstract

*De novo* or autosomal dominant *BAG3* gene variants cause a wide range of skeletal and cardiac muscle diseases encompassing Charcot–Marie–Tooth disease, myofibrillar myopathy, cardiomyopathy or a combination of them. Given the severity and rarity of *BAG3*-neuromuscular diseases (NMDs), series of patients are lacking. Our aim was to characterize the clinical and genetic spectrum, in addition to the natural history of *BAG3*-NMDs in Europe.

In this multicentre retrospective study, we collected clinical, ancillary and genetic data of patients with NMD and *BAG3* variants, identified from European paediatric and adult neuromuscular reference centres from May to December 2023 following a call circulated through the European Reference Network EURO-NMD and other partners. Responses were received from 35 centres in 17 countries. Twenty-six patients (65.4% males, 34.6% females) with *BAG3-*NMD from 18 different families were included in the study. The c.626C>T p.(Pro209Leu) variant, carried by 16 patients, was the only recurrent variant and was associated with a homogeneous and severe phenotype, with predominantly lower limb motor weakness (*n* = 13, 81.25%) or heart failure (*n* = 3, 18.75%) as the presenting feature and with a mean age at symptom onset of 7.8 ± 3.4 years. Where available (*n* = 13), electroneuromyography showed a polyneuropathy with demyelinating features and a frequently associated myopathy. Eleven (68.8%) patients had restrictive cardiomyopathy on initial assessment. Orthopaedic manifestations were common, with contractures (*n* = 15, 93.8%), foot deformities (*n* = 11, 84.6%) and scoliosis and/or rigid spine (*n* = 12, 80%). At last follow-up (age 21.5 ± 8.6 years) of the patients carrying the p.(Pro209Leu) variant, 10 (62.5%) had lost ambulation, 14 (93.3%) had respiratory insufficiency (11 requiring ventilation) and 12 (75%) had a restrictive cardiomyopathy, leading to heart failure and heart transplantation in five and four patients, respectively. Eight (50%) patients died prematurely at a mean age of 22.5 ± 9.6 years, most frequently from sudden death (*n* = 5). The other 10 patients carried three other *BAG3* variants and showed a milder disease course, with all patients remaining ambulatory, without cardiorespiratory manifestations at last follow-up. The p.(Arg309*) nonsense variant, known to cause isolated dilated cardiomyopathy, and the p.(Val505Glyfs*6) frameshift variant, resulting in a premature stop codon, caused distal hereditary motor neuropathy.

This is the largest study of patients with *BAG3*-NMD, delineating the frequency, specific presentation and natural history in patients with the recurrent *BAG3* p.(Pro209Leu) missense variant, crucial for informing patient management in the context of a rapidly progressive disease. All other *BAG3* variants were rare and caused milder clinical presentations.

## Teaser

Fernández-Eulate *et al.* describe the clinical and genetic spectrum, along with the natural history, of *BAG3*-related neuromuscular diseases in Europe. They show that the recurrent p.(Pro209Leu) variant causes severe, paediatric-onset myopathy, neuropathy, and cardiomyopathy, whereas other variants result in milder manifestations.

## Introduction

BCL2-associated athanogene (BAG) proteins are a family of anti-apoptotic co-chaperone proteins sharing a common BAG domain, which binds to the chaperone heat-shock protein 70 (HSP70) system^1^ to participate in protein quality control. BAG3 is expressed predominantly in skeletal and cardiac muscle and plays a role in the structural integrity of the muscle Z-disc^2^ and the cellular resilience to mechanical stress through the chaperone-assisted selective autophagy (CASA) complex, which participates in the lysosomal degradation of misfolded proteins and protein aggregates.^3,4^

Autosomal dominant variants in the *BAG3* gene are associated with dilated cardiomyopathy (DCM) (OMIM #613881).^5^ Less frequently, *de novo* or autosomal dominant variants can be associated with a wider spectrum of neuromuscular diseases (NMDs) encompassing myofibrillar myopathy (MFM) (OMIM #612954) and Charcot–Marie–Tooth disease (CMT).^6,7^

Several *BAG3* gene variants causing NMD have been described, but case reports suggest that the c.626C>T p.(Pro209Leu) variant in particular might be associated with a severe form of MFM, which may include a neuropathy and orthopaedic, cardiac and respiratory manifestations.^6^ Based on the scarce information currently available, the disease appears to be rapidly progressive. In addition to patients with the p.(Pro209Leu) variant, there are case reports of very rare *BAG3* variants associated with more heterogeneous NMD phenotypes, such as CMT type 2^7^ and distal hereditary motor neuropathy (dHMN).^8^

Currently, no treatments are approved for the neuromuscular manifestations of *BAG3*-related disease, but several therapies are at a preclinical development stage. Metformin has been shown to rescue muscle function in a zebrafish model of *BAG3* MFM,^9,10^ and a mouse model replicating key pathophysiological features of *BAG3* p.(Pro209Leu) patients has helped to explore the underlying molecular disease mechanisms and suggest a rescue strategy,^11,12^ providing the basis for the development of adeno-associated virus-mediated gene therapy and RNA interference approaches in the future.

With the aim of understanding the full genetic spectrum and associated clinical presentations of *BAG3*-NMD and to inform patient care and clinical trial design, we collected demographic, baseline and follow-up data of a large European cohort of patients with NMD and *BAG3* variants. We then described the clinical and genetic features, including tentative genotype–phenotype correlations, and studied the natural history and long-term prognosis of the disease.

## Materials and methods

### Study design and population

This was a multicentre retrospective study reviewing the clinical, genetic and long-term follow-up data of patients with *BAG3*-related NMD. To identify patients for the study, we used two different strategies: (i) a call was circulated in May 2023 through the European Reference Network for Neuromuscular disease (ERN EURO-NMD) to paediatric and adult neuromuscular reference centres in Europe; and (ii) e-mail invitations were sent individually to 41 different centres in Europe between May and December 2023.

The inclusion criteria were: (i) evidence of neuromuscular disease clinically or in electroneuromyography (ENMG) or muscle histology; and (ii) presence of a pathogenic or likely pathogenic *BAG3* variant.

The study was approved by the Research Ethics Committee of Sorbonne Université on 13 April 2023.

### Clinical and ancillary data

Anonymized data from clinical records were collected through a structured survey. The following data were collected: first symptom, age at onset, age at first examination, neurological examination findings [including Medical Research Council (MRC) scale], initial creatine kinase (CK) level (normal < 200 U/l), ENMG findings with detailed nerve conduction study data, lower limb or whole-body muscle MRI, including T1-weighted and short-tau inversion recovery (STIR) sequences, histological results from muscle biopsies, age at last follow-up and ambulatory status (including the need for a walking aid or loss of ambulation). Cardiac (symptoms, ECG, cardiac echography and/or MRI, cardiac events), orthopaedic (contractures, foot deformities, rigid spine, scoliosis, orthopaedic surgery) and respiratory assessments (last forced vital capacity in pulmonary function tests, use of assisted ventilation) were also collected, in addition to age at and cause of death.

### Genetics

*BAG3* (NM_004281.4, NP_004272.2) variants were identified by Sanger sequencing, a dedicated next-generation sequencing gene panel or through whole-exome sequencing. Variants were interpreted according to the recommendations of the American College of Medical Genetics; i.e. variant type and localization, specific protein domain, allelic frequency in the GnomAD v.4 exome database, combined annotation-dependent depletion (CADD) tool score, SpliceAI/SPiP tool to predict the effect of the variant on mRNA splicing, and the Leiden Open Variation Database 3.0 (LOVD3), Human Gene Mutation Pro Database (HGMD Pro). After interpretation, the variants were classified according to the American College of Medical Genetics classification criteria: i.e. Class 5 for pathogenic variants, Class 4 for likely pathogenic variants and Class 3 for variants of unknown significance.

### Statistical analysis

Data analyses were performed with SAS v.9.4 software, and all *P*-values < 0.05 were considered statistically significant. The number and proportion of available data were described for each variable. Qualitative variables were described in terms of numbers and proportions. Quantitative variables were described in terms of numbers, mean, standard deviation, median, minimum, maximum and interquartile range. Comparisons between groups were performed: (i) using χ² or Fisher tests depending on the values of the numbers expected under the assumption of independence between qualitative variables; or (ii) using Student’s or Mann–Whitney–Wilcoxon tests depending on the distribution of the variable of interest (between qualitative variables and quantitative variables). Survival analyses were performed using Kaplan–Meier curves and compared using the log-rank test.

Using R programming language, v.4.3.0, a hierarchical clustering analysis was performed to explore patterns of muscle involvement across patients based on Mercuri MRI scores. Muscle-specific metadata (e.g. age at symptom onset, time from onset to MRI and loss of ambulation at MRI) were included as annotations and visually encoded with colour gradients or discrete palettes. Two-dimensional hierarchical clustering was applied using ‘Complete’ as the linkage criterion and the ‘Manhattan distance’ as the dissimilarity metric. This combination was chosen based on its robustness for detecting compact and spherical clusters in clinical data with moderate noise and potential collinearity. All heatmaps were rendered using the ComplexHeatmap package. The Spearman correlation was used to study the correlation between disease duration (‘Onset to MRI’) and muscle involvement on MRI.

## Results

### Patient series and genetics

**Figure 1:**
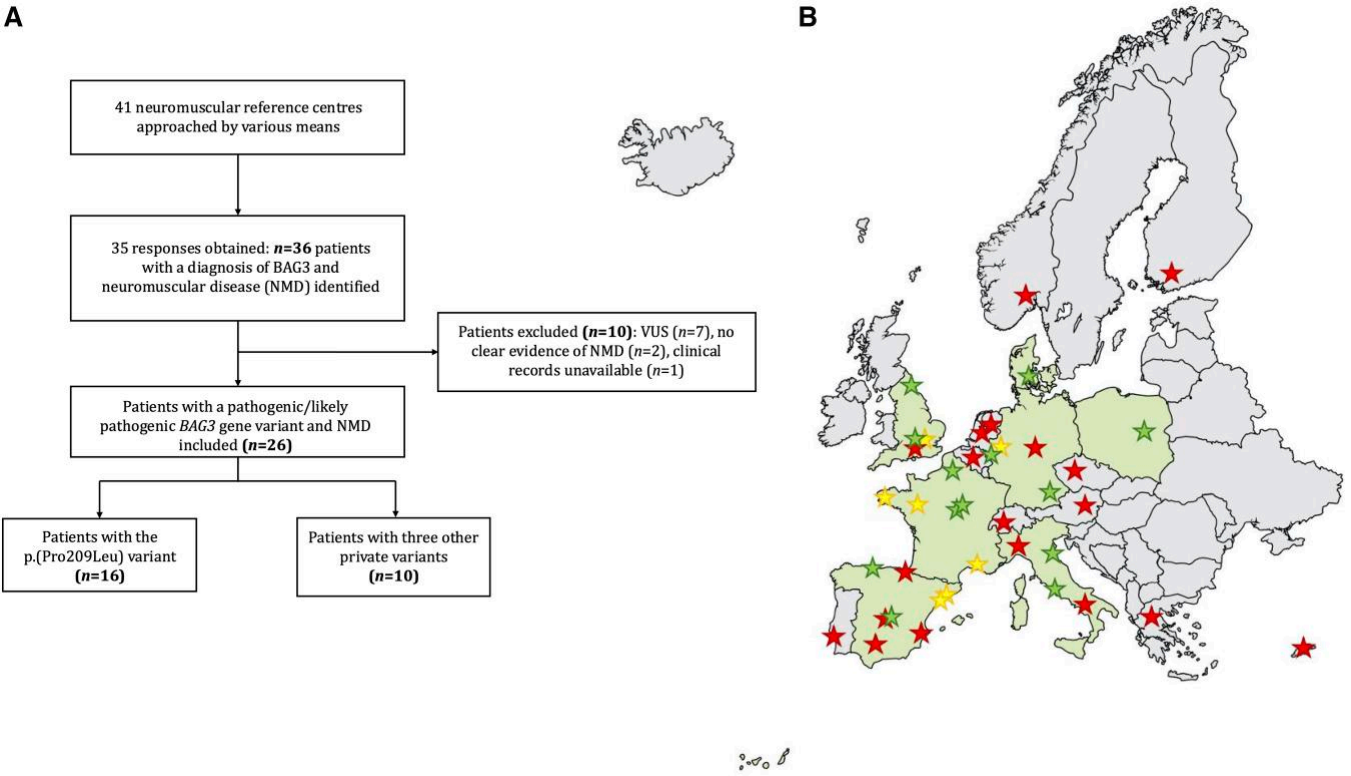
**Flowchart of the study and the *BAG3*-NMD population.** (**A**) Flowchart of the study. (**B**) Map of Europe depicting centres that responded to the call. Red stars indicate centres that reported not having any *BAG3*-NMD patients, yellow stars indicate centres with patients who were identified but not included in the study, and green stars indicate the centres with *BAG3*-NMD patients who were finally included in the series. The figure was partly created in BioRender. Fernández, G. (2025) https://BioRender.com/pxrucb1. ERN EURO-NMD = European Reference Network for neuromuscular diseases; NMD = neuromuscular disease; VUS = variant of unknown significance.

Thirty-five centres (paediatric and adult) from 17 European countries (Austria, Belgium, Czech Republic, Cyprus, Denmark, Finland, France, Germany, Greece, Italy, the Netherlands, Norway, Poland, Portugal, Spain, Switzerland and the UK) responded to our call, resulting in the identification of 36 candidate patients with *BAG3* variants and suspected NMD. Ten patients were excluded: seven carried a variant of unknown significance, two did not have clear evidence of NMD clinically or on ancillary investigations, and the clinical records of one patient were unavailable. Thus, 26 patients with NMD and *BAG3* variants were finally included and were from Denmark, France, Germany, Italy, Poland, Spain and the UK (Fig. 1). Data from 17 of these patients (FR01, UK01, UK02, UK03, DE02, DK01, IT01, IT02, PL01 and ES02–ES09) had previously been published;^8,13-20^ however, we present here a more comprehensive description of their phenotype and additional follow-up data.

Sixteen patients from 15 families carried the common p.(Pro209Leu) variant, while 10 patients from three families carried three additional private variants, defined as rare gene variants, each of which is found in only a single family or a small population (Supplementary Table 1). In the p.(Pro209Leu) group, *de novo* inheritance was confirmed in 11 patients through Sanger sequencing (*n* = 10) or whole-exome sequencing (*n* = 1); in two previously reported siblings from one family, parental somatic mosaicism was responsible for an apparently recessive pedigree;^19^ and in three additional patients genetic analysis of the parents was not performed. In the patients carrying one of the other three *BAG3* variants, p.(Arg309*) and p.(Val505Glyfs*6) were inherited in an autosomal dominant manner, whereas p.(Pro209Gln) was a *de novo* variant.

### Baseline characteristics

The baseline characteristics of *BAG3*-NMD patients are shown in Table 1. Given the differential clinical presentation observed in patients carrying the p.(Pro209Leu) variant and those carrying the other three *BAG3* variants, the baseline characteristics of each of these two genetic subgroups are presented separately.

**Table 1:** Demographic and baseline features of the series

Feature	All (*n* = 26)	p.(Pro209Leu) (*n* = 16)	Other variants (*n* = 10)	*P*-value
Age at first examination, years, mean ± SD	29.1 ± 23.3	14.2 ± 6.5	53 ± 20.2	**<0.001**
Male, *n* (%)	17 (65.4)	10 (62.5)	7 (70)	0.696
Age at onset of first symptom, years, mean ± SD	19.4 ± 18.4	7.8 ± 3.4	40.1 ± 15.6	**0**.**004**
First symptom	–	–	–	0.177
Motor weakness, *n* (%)	22 (84.6)	13 (81.25)	9 (90)	–
Heart failure, *n* (%)	3 (11.5)	3 (18.75)	0	–
No symptoms, *n* (%)	1 (3.8)	0	1 (10)	–
Facial weakness, *n* (%)^a^	0	0	0	–
Axial weakness, *n* (%)^a^	9 (4)	9 (60)	0	**0**.**008**
Scapula alata, *n* (%)^a^	7 (30.4)	7 (53.8)	0	**0**.**005**
Motor weakness, *n* (%)	–	–	–	**0**.**029**
Predominantly distal	15 (57.7)	6 (37.5)	9 (90)	–
Predominantly proximal	5 (19.2)	5 (31.3)	0	–
No predominance	3 (11.5)	3 (18.8)	0	–
Unknown distribution	2 (7.7)	2 (12.5)	0	–
Normal, only muscle cramps	1 (3.8)	0	1 (10)	–
Impaired pinprick sensation, *n* (%)^a^	3 (13.6)	1 (8.3)	2 (20)	0.427
Impaired vibration/proprioception, *n* (%)^a^	11 (52.4)	7 (63.6)	4 (40)	0.279
Absent deep tendon reflexes, *n* (%)^a^	16 (80)	10 (90.9)	6 (66.7)	0.178
Scoliosis and/or rigid spine, *n* (%)^a^	13 (52)	12 (80)	1 (10)	**<0**.**001**
Contractures, *n* (%)	12 (57.7)	15 (93.8)	0	**<0**.**001**
Foot deformities, *n* (%)^a^	12 (52.2)	11 (84.6)	1 (10)	**<0**.**001**
Creatine kinase, U/l, mean ± SD^a^	592.3 ± 428.4	636.5 ± 396	531.4 ± 490.4	0.443
Restrictive cardiomyopathy^a^	11 (50)	11 (68.8)	0	**0**.**004**

Patients were divided into those carrying the p.(Pro209Leu) variant and those carrying other *BAG3* variants; statistical analyses were performed between these two groups; *P*-values < 0.05 are shown in bold. SD = standard deviation.

^a^The percentage is calculated according to the number of observations.

#### Patient group carrying the p.(Pro209Leu) variant

**Figure 2:**
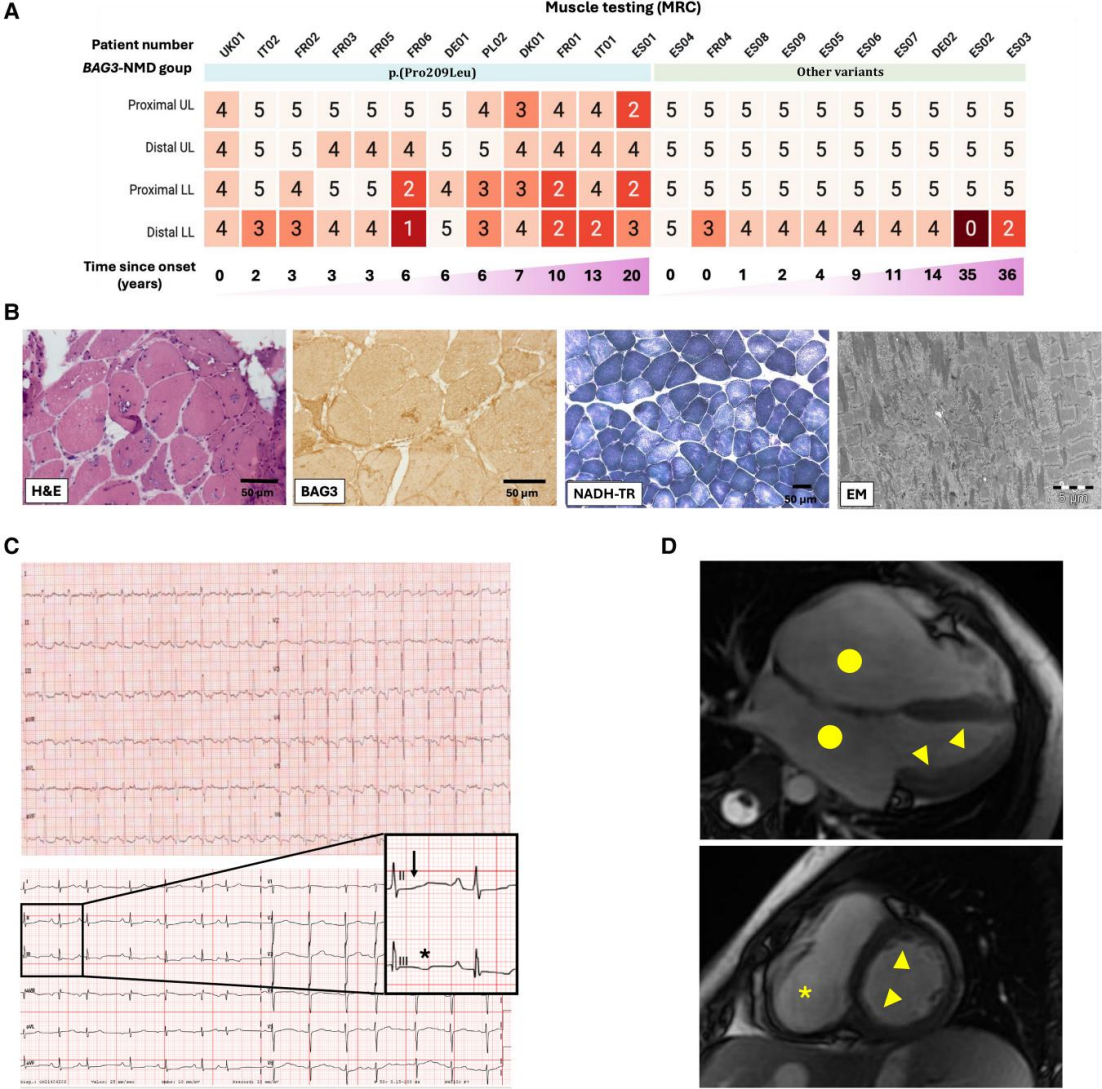
**Clinical presentation of *BAG3*-NMD patients.** (**A**) Heat map of muscle testing using the MRC sum score (0–5) for proximal versus distal, and upper versus lower limb muscles in patients from the p.(Pro209Leu) group versus those carrying other *BAG3*-NMD variants; patients are ordered according to increasing time since onset of disease to muscle testing evaluation. (**B**) Muscle histology of p.(Pro209Leu) patients shows myopathic changes and rimmed vacuoles (haematoxylin and eosin staining), BAG3 deposits (BAG3 stain) and myofibrillar disorganization (NADH-TR and electron microscopy). (**C**) ECGs showing negative T waves (star-shaped symbol) and ST segment depression (arrow) in the apical, inferior and lateral leads in two p.(Pro209Leu) patients, the first with evidence for a restrictive cardiomyopathy on cardiac imaging, whereas in the second cardiac imaging was normal. (**D**) Cardiac MRI long-axis (4-chamber apical view) and short-axis views (*top* and *bottom* panels, respectively) from a p.(Pro209Leu) patient showing left and right atrial enlargement (circle), global left ventricular wall thickening (arrowheads) and right ventricular dilatation and thickening (star-shaped symbol). The figure was partly created in BioRender. Fernández, G. (2025) https://BioRender.com/ypv6ikj. LL = lower limbs; NMD = neuromuscular disease; UL = upper limbs.

Patients carrying the p.(Pro209Leu) variant (*n* =16) had severe neuropathy and myopathy, with an onset in childhood (mean age: 7.8 ± 3.4 years) characterized by lower limb motor weakness as the first symptom in 81.25% of patients and by heart failure in the rest of the patients. At first examination (14.2 ± 6.5 years), motor weakness was distal in 37.5% of patients and proximal in 31.3%, with frequently associated axial weakness (60%) and scapula winging (53.8%). Where available, MRC muscle testing for proximal and distal upper and lower limb muscles is shown in Fig. 2A. Although impaired vibration sense and/or impaired proprioception were frequent (63.6%), superficial sensory impairment was observed in only one patient. Deep tendon reflexes were absent in most cases (90.9%). Orthopaedic features occurred frequently, with contractures present in 93.8% of patients, foot deformities (pes cavus with or without equinovarus) in 84.6%, and scoliosis and/or rigid spine in 80%. Blood CK levels were elevated (636.5 ± 396 U/l).

Where available (*n* = 13), ENMG findings were compatible with an intermediate form of neuropathy in five patients, axonal neuropathy in four patients, and mixed myopathic and neuropathic phenotype in another four patients, with myopathic motor unit potentials predominating in proximal muscles and neuropathic motor unit potentials in distal muscles (Table 2). Detailed nerve conduction study data were available in 10 of these patients (Supplementary Table 2), confirming the systematic observation of a sensory and motor neuropathy, with intermediate motor nerve conduction velocities in the median nerve (defined as motor nerve conduction velocity = 35–45 m/s in the context of ≥50% of normal median or ulnar nerve compound motor action potential) in seven patients (70%).

**Table 2:** Summary of the neurophysiological results in *BAG3*-NMD patients

ENMG findings	p.(Pro209Leu) (*n* = 13)	Other variants (*n* = 9)
**ENMG diagnosis**		
Intermediate CMT	5	0
Axonal CMT	4	0
Mixed CMT + myopathy	4	1
Myopathy	0	0
HMN	0	7
Normal	0	1
**NCS**	**(*n*** = **10)**	**(*n*** = **8)**
Age at examination, years, mean ± SD	13.7 ± 5.7	51.7 ± 17.8
Median CMAP, mV, median (range)	5.8 (0.1–9.9)	9.5 (6–12.8)
MNCV, m/s, median (range)	43.4 (34–46)	58 (53–64)
Ulnar CMAP, mV, median (range)	3.3 (0.4–7)	11.8 (8.9–14–6)
MNCV, m/s, median (range)	41 (34–42)	63.5 (58–69)
Fibular CMAP, mV, median (range)	0.4 (0–2.1)	5.4 (0.2–7.8)
MNCV, m/s, median (range)	30 (29–35)	46.5 (38–61.1)
Median SNAP, µV, median (range)	3.5 (0–10.6)	28.7 (14.8–40.5)
Sural SNAP, µV, median (range)	0 (0–0.6)	10.5 (6.5–22.4)

The first electroneuromyograph (ENMG) (or the ENMG closest to the first clinical examination) was studied, and the best side (in terms of amplitude of CMAP or SNAP) of each nerve is shown. A neuropathic ENMG was defined as large-amplitude and long-duration motor unit potentials and decreased motor unit potential recruitment. A myopathic ENMG was defined as short-duration and low-amplitude motor unit potential with rapid recruitment. CMAP = compound motor action potential (in millivolts); CMT = Charcot–Marie–Tooth disease; HMN = hereditary motor neuropathy; MNCV = motor nerve conduction velocity (in metres per second); NA = not available; NCS = nerve conduction studies; NMD = neuromuscular disease; SNAP = sensory nerve action potential (in microvolts).

**Figure 3:**
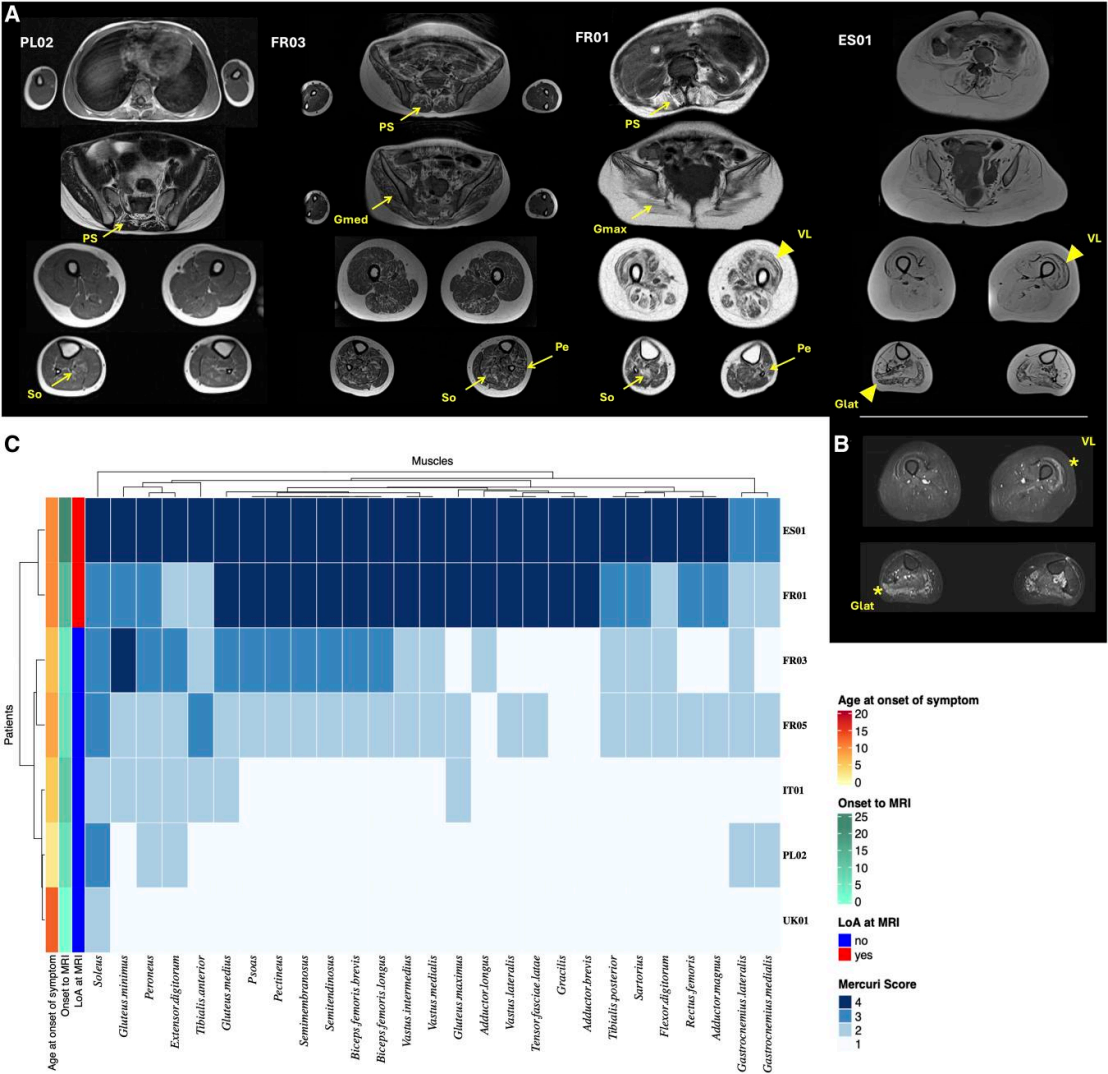
**Muscle MRI in p.(Pro209Leu) *BAG3* patients.** (**A**) Muscle MRI T1-weighted sequences of Patients PL02 (10 years old, with preserved independent ambulation), FR03 (11 years old, loss of independent ambulation), FR01 (23 years old, complete loss of ambulation) and ES01 (32 years old, complete loss of ambulation). PL02 shows mild increased signal intensity suggestive of fatty transformation of paraspinal muscles and soleus muscles. FR03 shows moderately increased signal intensity of paraspinal and glutei muscles, muscles of the thigh (with a predominance in the posterior compartment) and the legs (particularly soleus and peroneus muscles). FR01 shows severe increased signal intensity of paraspinal and glutei muscles, muscles of the thigh with relative vastus lateralis sparing (arrowhead) and more mild leg muscle involvement, predominating in the peroneus and soleus muscles. ES01 shows diffuse end-stage increased signal intensity of paraspinal, glutei and lower limb muscles with relative sparing of vastus lateralis and gastrocnemius muscles. (**B**) Example of short-tau inversion recovery (STIR) hyperintensities in relatively spared muscles (stars) in patient ES01. (**C**) A heat map representing the Mercuri scores of muscle involvement on MRI, with colours ranging from light blue (1) to dark blue (4); columns are labelled with muscle names, and rows are labelled with patient identifiers. Glat = gastrocnemius lateralis; Gmax = gluteus maximus; Gmed = gluteus medius; LoA = loss of ambulation; PS = paraspinal; Pe = peroneus; So = soleus; VL = vastus lateralis.

Muscle MRI (*n* = 7) (Fig. 3) showed systematic soleus muscle involvement, with associated early involvement of the anterior compartment of the leg (peroneus, extensor digitorum and tibialis anterior muscles), gluteus and posterior compartment of the thigh (semimembranosus, semitendinosus and biceps femoris muscles). Interestingly, gastrocnemius muscles were relatively preserved until very late stages of the disease. Additionally, STIR hyperintensities were frequent in muscles that were relatively spared, most likely accounting for muscle oedema in the context of degeneration and denervation. Importantly, the severity of muscle involvement in MRI was correlated with disease duration (‘Onset to MRI’), and this was particularly strong for the distal lower limb muscles and the gluteal muscles (Spearman’s ρ = 0.70; *P* = 0.08 for both muscle groups).

Muscle histology (*n* = 10) (Fig. 2B) revealed myopathic changes in all patients, with internalized nuclei (and even centralized nuclei in four cases), variation in fibre size and atrophic fibres. Vacuoles were observed in four cases; necrosis was not mentioned in any histology report, and endomysial fibrosis, if present, was generally mild to moderate. Alterations of myofibrillar structure were present in all biopsies, both in the form of myofibrillar disorganization in NADH staining and the presence of minicore or core-like structures in cytochrome *c* oxidase staining. Furthermore, aggregates were observed in nine patients. A positive desmin immunostaining was observed in all patients tested (*n* = 5), and αβ-crystallin and BAG3 immunostaining, each performed in one patient, were positive in both cases. Importantly, in seven cases (70%) clear neuropathic changes were observed, with angular fibres and fibre type grouping.

Notably, 68.8% of patients had findings compatible with a restrictive cardiomyopathy pattern at first examination on cardiac ultrasound or MRI, and a specific ECG pattern was identified, characterized by repolarization abnormalities, including negative T waves and ST segment depression, in the apical, inferior and lateral leads in all of the patients whose ECG could be re-examined (*n* = 7) (Fig. 2C).

#### Patient group carrying other *BAG3*-NMD variants

Patients carrying other BAG3-NMD variants (*n* = 10) had pure distal motor weakness, with a significantly later onset of disease (40.1 ± 15.6 years) in 9 of 10 patients. The 10th patient was a member of a large Spanish family carrying the p.(Val505Glyfs*6) variant and experienced no weakness but frequent muscle cramps, had a normal ENMG examination, and signs of reinnervation were present on tibialis anterior muscle biopsy. None of these patients had axial weakness, and orthopaedic features were very rare, with only one patient presenting with spinal rigidity and foot equinovarus deformities. No other contractures were seen. Similarly to patients carrying the p.(Pro209Leu) variant, only two patients had impaired pinprick sensation and four impaired vibration sensation on clinical examination. The CK values were also elevated (531.4 ± 490.4 U/l). Where available (*n* = 9), ENMG findings were compatible with dHMN in seven patients, one had a mixed axonal neuropathy and myopathy, and one case was normal, as mentioned above (Table 2). Detailed nerve conduction study data were available in eight patients (Supplementary Table 2), confirming normal nerve conduction velocities in all the nerves examined.

In summary, when compared with *BAG3*-NMD patients carrying any of the other three *BAG3*-NMD variants, p.(Pro209Leu) patients had a significantly earlier onset of disease (7.8 ± 3.4 versus 40.1 ± 15.6 years, *P* = 0.004), higher frequency of proximal or proximodistal motor weakness (50% versus none, *P* = 0.029), axial weakness (60% versus none, *P* = 0.008), scapular winging (53.8% versus none, *P* = 0.005), orthopaedic features including contractures (93.8% versus none, *P* < 0.001), foot deformities (84.6% versus 10%, *P* < 0.001) and scoliosis and/or rigid spine (80% versus 10%, *P* < 0.001), and restrictive cardiomyopathy (68.8% versus none, *P* = 0.004).

### Long-term outcomes

The analysis of long-term outcomes over a mean disease duration of 13.6 ± 9.6 years and a mean age at last follow-up of 34 ± 21 years indicated that disease progression was also substantially different between the two genetic subgroups, with the p.(Pro209Leu) group showing more pronounced morbidity and mortality (Table 3).

**Table 3:** Long-term outcomes in *BAG3*-neuromuscular disease patients

Outcome	All (*n* = 26)	p.(Pro209Leu) (*n* = 16)	Other variants (*n* = 10)	*P*-value
Age at last follow-up, years, mean ± SD	34 ± 21	21.5 ± 8.6	53.9 ± 19.5	**<0.001**
Time from onset of symptoms, years, mean ± SD	13.6 ± 9.6	13.8 ± 7.4	13.4 ± 13.2	0.556
Complete loss of ambulation, *n* (%)	10 (38.5)	10 (62.5)	0	**0.001**
Respiratory insufficiency, *n* (%)^a^	14 (56)	14 (93.3)	0	**<0.001**
Ventilation, *n* (%)	11 (42.3)	11 (68.8)	0	**<0.001**
Non-invasive ventilation	6 (23.1)	6 (37.5)	–	–
Tracheostomy	5 (19.2)	5 (31.3)	–	–
Restrictive cardiomyopathy, *n* (%)	12 (54.5)	12 (75)	0	**0.002**
Heart failure^a^	5 (20)	5 (33.3)	–	–
Cardiac transplantation	4 (15.4)	4 (25)	–	–
Scoliosis surgery	3 (11.5)	3 (18.8)	0	0.145
Deceased, *n* (%)	9 (34.6)	8 (50)	1 (10)	**0.037**
Sudden death	5 (19.2)	5 (31.3)	0	–
Respiratory insufficiency	1 (3.8)	1 (6.3)	0	–
Heart failure	1 (3.8)	1 (6.3)	0	–
Other	1 (3.8)	0	1 (10)	–
Unknown	1 (3.8)	1 (6.3)	0	–

Patients were divided into those carrying the p.(Pro209Leu) variant and those carrying other *BAG3* variants; statistical analyses were performed between these two groups; *P*-values < 0.05 are shown in bold. SD = standard deviation.

^a^The percentage is calculated according to the number of observations.

Of the 16 patients in the p.(Pro209Leu) group, 10 (62.5%) lost ambulation at a mean age of 18.6 ± 5.9 years. Three of these patients underwent surgery for rapidly progressive scoliosis, and in two further patients, surgery suggested for the same reason was not considered safe owing to a very high cardiorespiratory perioperative risk. In this respect, 93.3% of patients in this group had respiratory insufficiency, with 37.5% requiring non-invasive ventilation and 31.3% needing tracheostomy for invasive ventilation. The mean age at initiation of assisted ventilation was 16.2 ± 4.6 years. Pulmonary function tests (*n* = 9) at last assessment (mean age of 19.3 ± 8.2 years) showed a median forced vital capacity of 28% (range = 14%–87%). Among the seven patients without detailed pulmonary function tests, six had severe respiratory insufficiency requiring assisted ventilation, four of whom were invasively ventilated, and one died suddenly, shortly after diagnosis. Among the three patients with restrictive respiratory insufficiency who were not receiving assisted ventilation, two exhibited moderate respiratory insufficiency (last forced vital capacity: 54% and 61%), and one had severe respiratory insufficiency (last forced vital capacity: 28%). In this last patient, blood gas analysis and polysomnography did not show hypoventilation; however, she unfortunately suffered sudden death within the same year as the last evaluation.

A total of 12 (75%) *BAG3*-NMD patients, all of them carrying the p.(Pro209Leu) variant, developed a restrictive cardiomyopathy, associated with heart failure symptoms despite a preserved systolic function. Of these patients, five developed terminal heart failure at a mean age of 12.6 ± 2.6 years, which was lethal in one patient and required a cardiac transplantation in four at ages 8–14 years. Two of these patients died 11 and 25 years after the transplantation; the first was also under invasive ventilation and died suddenly, and the second one died following a brief intensive care unit admission, but no further details were available. The other two transplanted patients were still alive 4 and 8 years after the procedure. No evidence of arrhythmia or major conduction defects could be ascertained; however, five patients died suddenly of unknown cause, without recording of their cardiac rhythm during their cardiac arrest.

**Figure 4:**
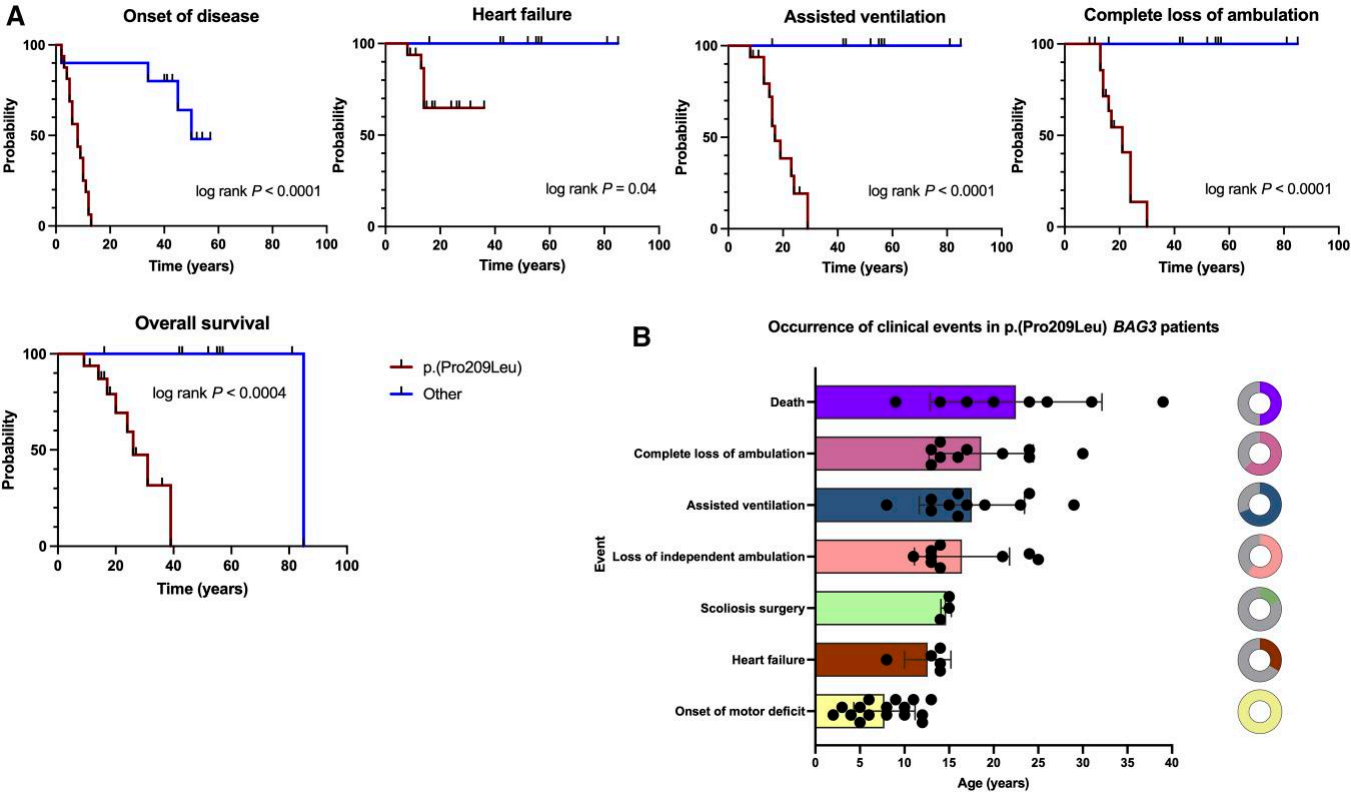
**Progression of disease in *BAG3*-neuromuscular disease patients.** (**A**) Kaplan–Meier and log rank test of the most important outcome events, including onset of disease, heart failure (and, in four cases, cardiac transplantation), assisted ventilation, complete loss of ambulation and overall survival. (**B**) Scatter plot of clinical events in p.(Pro209Leu) patients representing, for any given event, each single patient as a single point. Bars show mean age and whiskers the standard deviation. Doughnuts show the number of p.(Pro209Leu) patients with a given event out of the total number of patients for whom information was available for that event.

Importantly, heart failure and assisted ventilation occurred at an earlier median age than complete loss of ambulation (Fig. 4B), underpinning the severity of cardiorespiratory involvement in p.(Pro209Leu) *BAG3*-NMD.

In total, eight patients (50%) in the p.(Pro209Leu) group died prematurely at a mean age of 22.5 ± 9.6 years, predominantly from sudden death (*n* = 5), but also from respiratory insufficiency, heart failure or unknown cause (*n* = 1, respectively). The patients who suffered sudden death all had respiratory insufficiency (two under invasive and one under non-invasive ventilation); four had restrictive cardiomyopathy (one had a heart transplant), and four patients had lost ambulation. In one patient, sudden death occurred during an intercurrent respiratory infection.

In the group of patients carrying the other three BAG3 variants, none had lost ambulation and none showed signs of restrictive cardiomyopathy during follow-up. Furthermore, none showed signs of restrictive respiratory insufficiency and only one patient died, at 85 years old, from bilateral pneumonia in the context of chronic obstructive pulmonary disease.

Survival analysis confirmed significant differences between genetic subgroups with regard to the most important outcome events, including onset of disease (*P* < 0.0001), heart failure (*P* = 0.04), assisted ventilation (*P* < 0.0001), complete loss of ambulation (*P* < 0.0001) and overall survival (*P* < 0.0004) (Fig. 4A).

### Other genotype–phenotype considerations

**Figure 5:**
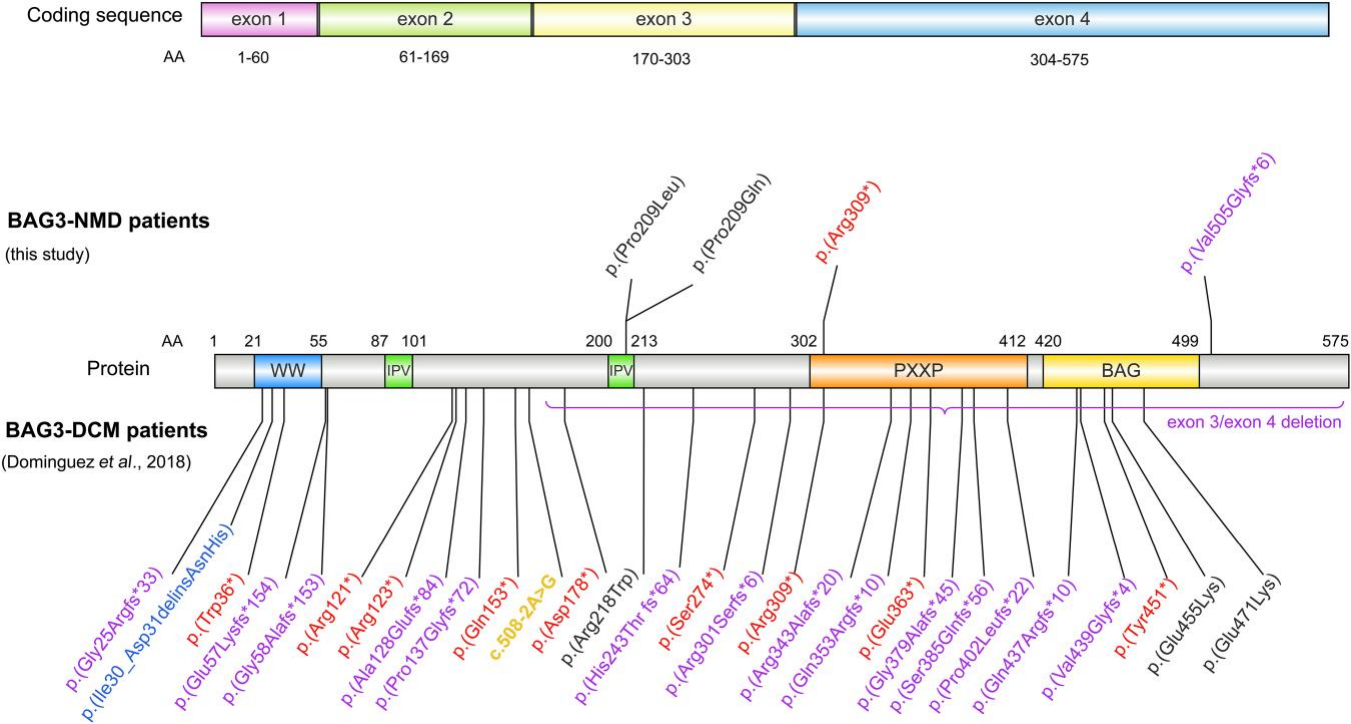
***BAG3* gene variants.** Schematic representation of the distribution on the BAG3 protein and nature of the gene variants identified in patients with neuromuscular disease in our study (top of the figure) versus those previously published by Domínguez *et al*.^5^ in patients with DCM in Europe (*bottom*). The main domains of the protein are indicated: the WW (tryptophan–tryptophan) domain, the two IPV (isoleucine–proline–valine) motifs, the PxxP (Pro-X-X-Pro) domain and the BAG domain. Missense variants are indicated in black, nonsense variants in red, splicing variants in orange, deletion–insertion variants in reading frame in blue, and variants resulting in a premature stop codon (frameshift following nucleotide insertions, deletions or duplications) in purple. DCM = dilated cardiomyopathy; NMD = neuromuscular disease.

The two missense and *de novo* variants [p.(Pro209Leu) and p.(Pro209Gln)] localized to the same amino acid (Pro209) in one of the two isoleucine–proline–valine motifs of the BAG3 protein, which bind HSPB1, HSPB5/αB-crystallin and HSPB8 small heat-shock proteins, were associated with a neuromyopathy. In contrast, the autosomal dominant p.(Val505Glyfs*6) variant, leading to a truncated BAG3 protein lacking the C-terminal LEAD sequence,^8^ and the p.(Arg309*) nonsense variant, associated with autosomal dominant DCM, both led to a dHMN phenotype (Fig. 5).

## Discussion

This study represents the largest series of patients with *BAG3*-NMD so far, depicting the full spectrum of clinical findings, genotype–phenotype correlations and natural history of the disease. Given the rarity of the disease, a Pan-European collaboration was essential to be able to gather sufficient data and present the most complete picture of the disease, at least in Europe. We show that the *de novo* p.(Pro209Leu) variant represents by far the most frequent *BAG3* variant and the only recurrent variant in our study of patients living in Europe. Furthermore, the disease presentation, severity and progression were substantially different in patients carrying the p.(Pro209Leu) variant compared with those carrying the other very rare *BAG3*-NMD variants.

### *BAG3*-NMD is a rare disease with two distinct genetic subgroups

The total estimated population in 2023 of the 17 European countries from which responses of at least one neuromuscular reference centre were obtained (Austria, Belgium, Czech Republic, Cyprus, Denmark, Finland, France, Germany, Greece, Italy, the Netherlands, Norway, Poland, Portugal, Spain, Switzerland and the UK) was 461 873 856 (https://data.worldbank.org). Of the 26 patients with *BAG3*-NMD included in our study, 17 patients are currently alive. Thus, the prevalence of *BAG3*-NMD can be estimated at 17/461,873,856 or ∼1 patient per 27 million people, in keeping with the definition of an ultra-rare disease, defined as a prevalence lower than 20 patients per million people.

Our study delineates a specific clinical and neurophysiological presentation and a different progression of disease in p.(Pro209Leu) patients when compared with patients carrying other *BAG3* variants. Patients carrying the p.(Pro209Leu) variant had a childhood-onset distal and proximal lower limb motor weakness associated with pes cavus, orthopaedic features, restrictive cardiomyopathy and respiratory insufficiency. This is similar to previously reported p.(Pro209Leu) patients from other areas of the world, although not all clinical manifestations may be concomitant at onset.^21-27^ Motor weakness in p.(Pro209Leu) patients is neuromyopathic, combining features of a motor and sensory polyneuropathy with intermediate nerve conduction velocities on nerve conduction study and mixed myopathic and neuropathic motor unit potentials on EMG.

Few case reports of muscle MRI of *BAG3*-NMD patients exist, but they support our findings that soleus involvement, followed by involvement of the gluteus and the posterior compartment of the thigh muscles, are an early and characteristic finding on muscle MRIs of patients carrying the p.(Pro209Leu) variant.^14,28^ Interestingly, soleus muscle involvement has also been observed in *BAG3*-NMD patients carrying other very rare variants, such as the p.(Pro209Ser) variant^29^ and the p.(Val505Glyfs*6) variant,^8^ and therefore this muscle MRI finding can help to guide the genetic diagnosis of *BAG3*-NMD, even in the context of different clinical presentations. However, in the latter *BAG3* variants, muscle involvement on MRI was clearly more distal.^8,29^ Importantly, this pattern is different from that of other types of hereditary peripheral neuropathy. CMT1A attributable to *PMP22* duplication is the most common form of CMT, and patients show early involvement of the anterior compartment of the leg and the gastrocnemius muscles, with sparing of the soleus and thigh muscles until late stages of the disease.^30^  *BAG3*-NMD muscle involvement on MRI would, in turn, resemble more closely that of pure motor neuropathies or dHMN, where soleus muscle involvement is an early phenomenon across most dHMN genes.^31^ However, gastrocnemius muscles are also frequently involved in dHMN and, as we show, are spared in *BAG3* patients. Furthermore, gluteus muscles are more frequently involved in *BAG3*-NMD patients in comparison to patients with other types of dHMN.^31^ Likewise, soleus involvement is frequently accompanied by gastrocnemius muscle involvement in MFM, and quadriceps muscle involvement predominates at the thigh level in several MFM causative genes,^32,33^ which is again different from the pattern observed in *BAG3*-NMD.

As previously mentioned, the p.(Pro209Leu) *BAG3* variant leads to neuropathy and myopathy, as is also the case for a number of other NMD genes,^34^ including the bicaudal Drosophila homologue 2 (*BICD2*),^35,36^ valosin-containing protein (*VCP*),^37^ small heat-shock proteins 1 and 8 (*HSPB1* and *HSPB8*)^38-41^ and, more recently, the von Willebrand Factor A domain containing 1 (*VWA1*) gene.^42^ The BAG3 protein is a co-chaperone implicated in multiple protein–protein interactions, including binding the HSP70 system and small heat-shock proteins (HSPB), and it plays a crucial role in protein quality control. It is thought that a combined mixed loss-of-function and dominant gain-of-function effect of the p.(Pro209Leu) *BAG3* variant, localized in the isoleucine–proline–valine motif, which binds the HSPB1, HSPB5/αB-crystallin and HSPB8 small heat-shock proteins, leads to the collapse of protein homeostasis and the accumulation of protein aggregates in patient muscle.^6,9,43^ Through defective axonal and vesicular transport (*BICD2*),^44^ alteration in the ubiquitin–proteasome system (*VCP*)^45,46^ or impaired chaperone-assisted selective autophagy or CASA (*HSPB1* and *HSPB8*),^47-49^ these neuromyopathy genes can also lead to a collapse of protein homeostasis and the accumulation of aggregates, which is therefore a common pathological mechanism in neuromyopathy genes.

Interestingly, both the nonsense p.(Arg309*) and p.(Val505Glyfs*6) frameshift *BAG3*-NMD variants were associated with dHMN, the latter variant being found in a large family from Spain with adult-onset dHMN.^8^ We now also report a patient with an early onset moderate dHMN, carrying the p.(Arg309*) nonsense *BAG3* variant, which has been reported previously in *BAG3*-DCM but never in association with NMD.^50^ In a series of 129 individuals with *BAG3*-DCM, mainly attributable to nonsense or frameshift variants,^5^ only one patient had a single increased CK value, in the context of heavy training, and the authors concluded that patients carrying *BAG3*-DCM gene variants did not seem to have any associated neuromuscular disease. However, no systematic assessment of the neuromuscular system was performed. Given that dHMN can be mild in many cases and that cardiac dyspnoea seen in *BAG3*-DCM can interfere with physical activity, we cannot exclude the possibility that other *BAG3* variants associated with DCM could also be responsible for dHMN or another mild neuromuscular disorder. Therefore, our findings suggest there should be a low threshold for a specialist neuromuscular assessment of patients with autosomal dominant *BAG3* variants primarily causing DCM.

To our knowledge, only two additional *BAG3* variants associated with NMD have been reported. The first one, a p.(Pro209Ser) variant, has been described in three families from the USA^7,51^ and one family from China.^29^ Intriguingly, this variant localizes to the same proline in position 209 as the p.(Pro209Leu) variant associated with the severe paediatric-onset neuropathy and myopathy with cardiac, respiratory and orthopaedic disease and the p.(Pro209Gln) variant present in one adult patient with a milder mixed axonal neuropathy and myofibrillar myopathy. However, the p.(Pro209Ser) variant is associated with a pure CMT2 phenotype and no overt cardiac disease.^7^ Another proline-to-serine change but localized to the 470 amino acid in the HSP70 binding region or BAG domain [p.(Pro470Ser) variant] has been reported in three additional families from the USA and Japan with milder neuromuscular disease.^43,52^

### The cardiomyopathy in p.(Pro209Leu) patients is restrictive and severe

Only *BAG3*-NMD patients carrying the p.(Pro209Leu) variant had cardiac manifestations, in the form of restrictive cardiomyopathy with preserved Left ventricular ejection fraction (LVEF) heart failure, which was present in more than half of the patients at first examination.

Therefore, the cardiac phenotype in *BAG3*-NMD p.(Pro209Leu) patients seems to be dominated by structural cardiac disease, as is the case in *BAG3*-DCM.^5^ Nevertheless, in contrast to autosomal dominant *BAG3*-DCM patients, p.(Pro209Leu) patients always showed restrictive cardiomyopathy. Why the p.(Pro209Leu) missense variant causes a different structural cardiac phenotype (in association with severe NMD) is unclear, because in both cases BAG3 protein levels seem to be diminished in cardiac tissue, with disorganized sarcomeric Z-discs.^5,53^ However, first, autosomal dominant *BAG3*-DCM^5^ is mostly associated with nonsense variants and frameshift variants, resulting in premature stop codons. Second, a possible associated dominant gain-of-function effect of the p.(Pro209Leu) missense variant has been reported,^43^ and third, this variant is located in one of the isoleucine–proline–valine motifs binding other NMD-causing proteins (like HSPB1 and HSPB5/αB-crystallin),^54^ which are generally spared in *BAG3*-DCM, and, taken together these factors could account for the differences in phenotype.

Importantly, we also showed that patients carrying the p.(Pro209Leu) variant present consistently with specific repolarization abnormalities, which could help in the diagnosis of *BAG3*-NMD attributable to this variant. The severity of cardiac disease is worth emphasizing, because appropriate screening for heart involvement is crucial to enable early institution of heart failure treatments. Two patients undergoing heart transplantation were still alive ≥4 years after the procedure, and the two deceased patients lived for >10 years after the transplantation, underlining that this treatment can be a successful option in patients with milder skeletal and respiratory involvement. Although no arrhythmia or major conduction defects were recorded in our patients, they might possibly have been overlooked in some cases, because sudden death was the most common mode of death in patients carrying the p.(Pro209Leu) variant. Sudden death can be a consequence of ventricular arrhythmias, major conduction defects or electromechanical dissociation in patients with restrictive cardiomyopathy, but might also be related to extracardiac causes, such as acute respiratory failure in the case of an unwitnessed arrest. Therefore, repeated monitoring of cardiac rhythm, at least annually, is strongly indicated in this population, with 24 h or longer duration Holter-ECG.

### Fast disease progression in p.(Pro209Leu) patients argues in favour of proactive and anticipatory management

As shown, the elapsed time between the onset of motor deficit and death is in many cases <15 years. Therefore, the time frame for making the correct management decisions, including spinal surgery and heart transplantation, is narrow. Furthermore, we have shown that cardiorespiratory involvement progresses more rapidly than motor weakness, with heart failure appearing at ∼12 years of age and patients needing assisted ventilation by the age of 17 years, whereas loss of ambulation occurred at a mean age of 18 years. Thus, *BAG3*-NMD caused by the p.(Pro209Leu) variant is similar to other neuromuscular diseases, such as laminopathies^55^ and desminopathies,^56^ insofar as cardiorespiratory involvement does not necessarily appear and/or progress in parallel with motor weakness. In our experience, orthopaedic features, and particularly scoliosis/rigid spine and contractures, also progress rapidly while the patient is still ambulant and, together with the frequently observed axial weakness, are likely to explain, in part, the severe respiratory insufficiency in this subgroup of patients. Therefore, ambulant p.(Pro209Leu) patients should be managed actively with anti-heart failure medication or even cardiac transplantation, scoliosis orthopaedic interventions, non-invasive and invasive ventilation and prevention of respiratory infections.

Our study has several limitations. Firstly, the data were collected retrospectively from many different centres across Europe. Nevertheless, we have managed to keep missing data to a minimum for the key variables and events, including the onset of symptoms, clinical presentation, cardiac and respiratory manifestations, loss of ambulation and death. Secondly, despite our efforts to reach as many neuromuscular units as possible, we may well have missed some *BAG3*-NMD patients for inclusion in our study. Thirdly, given the rarity of the disorder and the fact that variants other than p.(Pro209Leu) are generally private to a single family, it was not possible to conduct comparisons between individual variants. In this context and in the advent of new therapies and clinical trials in the disease, an international registry for patients with *BAG3*-NMD could be useful to identify more patients and better characterize the natural history of the disease.

## Conclusion

In conclusion, this is the largest series of patients with *BAG3*-NMD reported to date, with a wide spectrum of NMD presentations and emerging relevant genotype–phenotype correlations. The p.(Pro209Leu) variant causes severe myopathy and neuropathy with demyelinating features in combination with rapidly progressive orthopaedic and cardiorespiratory manifestations, indicating a narrow time frame to make the correct management decisions. These data should be useful for the design of future natural history studies and to inform patient management and clinical trials. All other *BAG3* variants are rare and cause milder clinical presentations, with nonsense variants and frameshift variants being associated with dHMN (and with isolated DCM), an observation which should be considered in the development of disease-modifying therapies.

## Supplementary Material

awaf223_Supplementary_Data

## Data Availability

The data that support the findings of this study are available from the corresponding author upon reasonable request.
