# Supplementary material for: Disease spectrum and long-term prognosis of patients with BAG3-associated neuromuscular diseases in Europe
Source: Brain. 2025 Jun 10;148(12):4435–47. doi: 10.1093/brain/awaf223 (PMC12678052; doi:10.1093/brain/awaf223)
Supplement: awaf223_Supplementary_Data [file awaf223_supplementary_data.pdf]

Supplementary table 1. Classification of *BAG3* variants in this study using different genetic criteria

Coding DNA reference (NM_004281.4)	Protein reference (NP_004272.2)	Variant type	Exon / Intron localisation	Protein domain	Allelic frequency gnomAD v4 exome	CADD	SpliceAI / SPiP	Clinvar	LOVD3	HGMD Pro	ACMG criteria	Patients (families) in this study, *n*	Transmission
c.626C>T[a]	p.(Pro209Leu)	Missense	3	IPV-motifs	No match	26.4	–	P	P	P	P	16 (15)	*De novo*
c.626C>A[b]	p.(Pro209Gln)	Missense	3	IPV-motifs	No match	26.6	Possible acceptor gain	P	NR	P	P	1	*De novo*
c.925C>T[c]	p.(Arg309*)	Nonsense	4	PxxP-domain	4.104×10^{-6}	40	–	P	NR	P	P	1	AD
c.1513_1514insGGAC[d]	p.(Val505Glyfs*6)	Nucleotide insertion	4	–	No match	–	–	NR	NR	NR	LP	8 (1)	AD

AD = autosomal dominant; LP = Likely pathogenic; NR = not reported; P = pathogenic.
[a]Publications: Selcen et al., 2009; Odgerel et al., 2010; Lee et al., 2012; Jaffer et al., 2012; Kostera-Pruszczyk et al., 2015; Konersman et al., 2015; Andersen et al., 2018; Noury et al., 2018; Kim et al., 2018; Schänzer et al., 2018; Malatesta et al., 2020; Scarpini et al., 2021; Xu et al., 2021; Akaba et al., 2022; Zhan et al., 2022; Butler-Browne et al., 2023 (Several patients from the present study previously published).
[b]Publications: Semmler et al., 2014 and Adriaenssens et al., 2020 (same patient).
[c]Publication: Stava et al., 2022.
[d]Publication: de Fuenmayor-Fernández de la Hoz *et al.,* 2024 (same family).

References:

1. Selcen D, Muntoni F, Burton BK, et al. Mutation in BAG3 causes severe dominant childhood muscular dystrophy. Ann Neurol. 2009;65(1):83-89. doi:10.1002/ana.21553
2. Odgerel Z, Sarkozy A, Lee HS, et al. Inheritance patterns and phenotypic features of myofibrillar myopathy associated with a BAG3 mutation. Neuromuscular Disorders. 2010;20(7):438-442. doi:10.1016/j.nmd.2010.05.004
3. Lee HC, Cherk SW, Chan SK, et al. BAG3-related myofibrillar myopathy in a Chinese family. Clin Genet. 2012;81(4):394-398. doi:10.1111/j.1399-0004.2011.01659.x
4. Jaffer F, Murphy SM, Scoto M, et al. BAG3 mutations: Another cause of giant axonal neuropathy. Journal of the Peripheral Nervous System. 2012;17(2):210-216. doi:10.1111/j.1529-8027.2012.00409.x
5. Kostera-Pruszczyk A, Suszek M, Płoski R, et al. BAG3-related myopathy, polyneuropathy and cardiomyopathy with long QT syndrome. J Muscle Res Cell Motil. 2015;36(6):423-432. doi:10.1007/s10974-015-9431-3
6. Konersman CG, Bordini BJ, Scharer G, et al. BAG3 myofibrillar myopathy presenting with cardiomyopathy. Neuromuscular Disorders. 2015;25(5):418-422. doi:10.1016/j.nmd.2015.01.009
7. Andersen AG, Fornander F, Schrøder HD, et al. BAG3 myopathy is not always associated with cardiomyopathy. Neuromuscular Disorders. 2018;28(9):798-801. doi:10.1016/j.nmd.2018.06.019
8. Noury JB, Maisonobe T, Richard P, Delague V, Malfatti E, Stojkovic T. Rigid spine syndrome associated with sensory-motor axonal neuropathy resembling Charcot-Marie-Tooth disease is characteristic of Bcl-2-associated athanogene-3 gene mutations even without cardiac involvement. Muscle Nerve. 2018;57(2):330-334. doi:10.1002/MUS.25631
9. Kim SJ, Nam SH, Kanwal S, et al. BAG3 mutation in a patient with atypical phenotypes of myofibrillar myopathy and Charcot–Marie–Tooth disease. Genes Genomics. 2018;40(12):1269-1277. doi:10.1007/s13258-018-0721-1
10. Schänzer A, Rupp S, Gräf S, et al. Dysregulated autophagy in restrictive cardiomyopathy due to Pro209Leu mutation in BAG3. Mol Genet Metab. 2018;123(3):388-399. doi:10.1016/J.YMGME.2018.01.001
11. Malatesta L, Arya K, Gokden M, Stefans V, Veerapandiyan A. BAG3 Myopathy Presenting With Prominent Neuropathic Phenotype and No Cardiac or Respiratory Involvement: A Case Report and Literature Review. J Clin Neuromuscul Dis. 2020;21(4):230-239. doi:10.1097/CND.0000000000000300
12. Scarpini G, Valentino ML, Giannotta M, et al. BAG3-related myofibrillar myopathy: a further observation with cardiomyopathy at onset in pediatric age. Acta Myol. 2021;40(4):177-183. doi:10.36185/2532-1900-061
13. Xu Y, Liu S, Xu W, et al. BAG3-Related Myofibrillar Myopathy Presenting as Hypercapnia: A Case Report and Literature Review. Chin Med Sci J. 2021;36(4):1. doi:10.24920/003883
14. Akaba Y, Takeguchi R, Tanaka R, et al. Wide Spectrum of Cardiac Phenotype in Myofibrillar Myopathy Associated With a Bcl-2-Associated Athanogene 3 Mutation: A Case Report and Literature Review. J Clin Neuromuscul Dis. 2022;24(1):49-54. doi:10.1097/CND.0000000000000392
15. Zhan L, Lv L, Chen X, Xu X, Ni J. Ultrasound evaluation of diaphragm motion in BAG-3 myofibrillar myopathy: A case report. Medicine. 2022;101(1):e28484. doi:10.1097/MD.0000000000028484
16. Butler-Browne S, Angelini C, Ceolin C, Rodriguez AA, Nigro V. Two Cases of Myofibrillar Myopathies: Genetic and Quality of Life Study. Muscles 2023, Vol 2, Pages 177-186. 2023;2(2):177-186. doi:10.3390/MUSCLES2020013

17. Semmler AL, Sacconi S, Bach JE, et al. Unusual multisystemic involvement and a novel BAG3 mutation revealed by NGS screening in a large cohort of myofibrillar myopathies. Orphanet J Rare Dis. 2014;9(1). doi:10.1186/s13023-014-0121-9
18. Adriaenssens E, Tedesco B, Mediani L, et al. BAG3 Pro209 mutants associated with myopathy and neuropathy relocate chaperones of the CASA-complex to aggresomes. Sci Rep. 2020;10(1). doi:10.1038/s41598-020-65664-z
19. Stava TT, Leren TP, Bogsrud MP. Molecular genetics in 4408 cardiomyopathy probands and 3008 relatives in Norway: 17 years of genetic testing in a national laboratory. Eur J Prev Cardiol. 2022;29(13):1789-1799. doi:10.1093/EURJPC/ZWAC102
20. de Fuenmayor-Fernández de la Hoz CP, Lupo V, Bermejo-Guerrero L, et al. Distal hereditary motor neuronopathy as a new phenotype associated with variants in BAG3. J Neurol. 2024;271(2):986-994. doi:10.1007/s00415-023-12039-9

Patient ID	Age at EMG (y)	Description	Motor (CMAP/MNCV)				Sensory (SNAP)				EMG
			**Median**	**Ulnar**	**Tibial**	**Fibular**	**Median**	**Ulnar**	**Sural**	**Superficial peroneal**	
**p.(Pro209Leu) patients (n=16)**											
FR01	20	CMT intermediate + myopathy	9,9/39	5,4/41	0,2/-	1,6/27	5,9	0	0	0	Myopathic (deltoid) and neuropathic with spontaneous activity (TA)
FR02	8	CMT intermediate	5.8/44	3.6/42	0.9/-	2.1/32	10.6	3.1	0	0	Neuropathic (TA)
FR03	10	CMT axonal	7.5/45		0.9/40	0.2/35	2.1				Neuropathic (quadriceps, TA and radial)
FR05	11	CMT axonal	6.2/46		0	0	3.4		0		Neuropathic (TA)
FR06	16	CMT intermediate	3.6/38	2.4/-		0.7/-	3				Neuropathic (quadriceps, TA, and IO)
UK01	12	CMT intermediate	0.3/-	2.9/36.5	0		4.5		0		Neuropathic with spontaneous activity (TA)
UK02		CMT axonal + myopathy									Neuropathic and myopathic
UK03		NA									
DE01		NA									
DK01		NA									
IT01		CMT axonal									
IT02	14	CMT intermediate + myopathy	8/34	7/42	0.5/30.8	0.5/30	1.15	0	0		Myopathic (deltoid and biceps brachii) and neuromyopathic with spontaneous activity (TA and medial gastrocnemius)
PL01	12	CMT intermediate		2.9/38		0.1/29	3.5	0	0		Neuropathic
PL02	8	CMT intermediate	3.8/42.8	3.7/41	0.2/43	0	7.5	4.8	0.6	0	Neuropathic
PL03	26	CMT axonal + myopathy	0.1/44	0.4/34			0	0			Myopathic (proximal) and neuropathic (distal)
ES01		CMT axonal									
**Other *BAG3* variants (n=10)**											
FR04	16	HMN	9/60.7		13.9/50.2	5.6/61.1	14.8		22.4		Neuropathic with spontaneous activity (TA)
DE02		CMT axonal + myopathy									
ES02	85	HMN	6.0/50			0.2/38	21		6.5		Neuropathic with spontaneous activity (TA)
ES03		NA									
ES04	57	Normal	9.5/53		8.6/45	7.4/49	22.2		17.2	7.4	Normal

ES05	56	HMN				7.8/45			11.3		Neuropathic (TA) with spontaneous activity (EDB)
ES06	52	HMN	12.3/59		1.3/43	7.4/48	38.3		15.6	22.4	Neuropathic (TA and EDB)
ES07	52	HMN				1.9/38			7.5		Neuropathic with spontaneous activity (TA)
ES08	55	HMN	9.4/64	8.9/69	1.4/41	5.2/49	40.5	59.8	9.5	6.3	Neuropathic (TA, gastrocnemius) with spontaneous activity (EDB, AHB)
ES09	42	HMN	12.8/57	14.6/58	1.2/41	0.4/42	35.1	37.7	9.7	6.0	Neuropathic with spontaneous activity (TA and gastrocnemius)

Supplementary table 2. Neurophysiological findings in BAG3-NMD patients. The first EMG (or closest to the first clinical examination) are presented and the best side (in terms of amplitude of CMAP or SNAP) of each nerve is shown. A neuropathic EMG is defined as large amplitude and long-duration Motor Unit Potentials (MUP) and decreased MUP recruitment. A myopathic EMG as short duration and low amplitude MUP with rapid recruitment. AHB = Abductor Hallucis Brevis; EDB = Extensor Digitorum Brevis; CMAP = compound motor action potential (in mV); CMT = Charcot-Marie-Tooth; HMN = Hereditary motor neuropathy; IO = interosseous; MNCV = motor nerve conduction velocity (in m/s); NA = not available; SNAP = sensory nerve action potential (in µV); TA = tibialis anterior; y = years.
